# Combined Treatments of Magnetic Intra-Lysosomal Hyperthermia with Doxorubicin Promotes Synergistic Anti-Tumoral Activity

**DOI:** 10.3390/nano8070468

**Published:** 2018-06-27

**Authors:** Darine El Hajj Diab, Pascal Clerc, Nizar Serhan, Daniel Fourmy, Véronique Gigoux

**Affiliations:** INSERM ERL1226—Receptology and Therapeutic Targeting of Cancers, Laboratoire de Physique et Chimie des Nano-Objets, CNRS UMR5215-INSA, Université de Toulouse III, F-31432 Toulouse, France; darine.diab@hotmail.fr (D.E.H.D.); pascal.clerc@inserm.fr (P.C.); nizar.serhan@inserm.fr (N.S.); daniel.fourmy@inserm.fr (D.F.)

**Keywords:** magnetic nanoparticles, magnetic hyperthermia, cell death, cancer, endocrine tumors, doxorubicin

## Abstract

Doxorubicin is a cytotoxic drug used for the treatment of many cancer types. However, its significant dose-related adverse effects including cardiotoxicity may hamper its efficiency. Moreover, the multidrug resistance that appears during treatments limits anti-cancer therapies. Hyperthermia has been introduced as an adjuvant anti-cancer therapy and presents promising opportunities especially in combination with chemotherapy. However, hyperthermia methods including standard magnetic hyperthermia do not discriminate between the target and the surrounding normal tissues and can lead to side effects. In this context, a Magnetic Intra-Lysosomal Hyperthermia (MILH) approach, which occurs without perceptible temperature rise, has been developed. We previously showed that minute amounts of iron oxide magnetic nanoparticles targeting the gastrin receptor (CCK2R) are internalized by cancer cells through a CCK2R-dependent physiological process, accumulated into their lysosomes and kill cancer cells upon high frequency alternating magnetic field (AMF) application through lysosomal cell death. Here, we show that the combination of MILH with doxorubicin increases the efficiency of the eradication of endocrine tumor cells with synergism. We also demonstrate that these two treatments activate two different cell death pathways that are respectively dependent on Caspase-1 and Caspase-3 activation. These findings will result in the development of new anti-tumoral, intra-lysosomal-thermo/chemotherapy with better curative effects than chemotherapy alone and that are devoid of adverse effects linked to standard hyperthermia approaches.

## 1. Introduction

Neuroendocrine tumors (NETs) are uncommon and heterogeneous neoplasms of epithelial origin, in which neoplastic cells retain many of the structural and functional characters of normal endocrine cells, including the expression of typical endocrine and neuroendocrine markers (such as chromogranin A and synaptophysin) and the capacity to synthesize, store and secrete peptidic hormones and neuropeptides [1]. Although NETs may develop in almost any organ, the predominate locations are within the pancreas and the gastrointestinal tract. The incidence of gastroenteropancreatic neuroendocrine tumors is 3.65/100,000 individuals per year which has increased markedly over the last 3 decades, probably as a result of trends in imaging and improvements in diagnosis. Although most endocrine tumors present slow and local development, some of them become malignant with metastatic disseminations and are associated with poor prognoses.

Conventional cancer therapies include surgery, radiotherapy and/or chemotherapy. Doxorubicin, which belongs to the anthracylin chemotherapeutic drug family, has been used for decades as a cytotoxic drug for the treatment of many cancer types, such as breast, lung, stomach, bone cancers and endocrine tumors, including gastroenteropancreatic neuroendocrine tumors [2]. Doxorubicin exerts its anti-tumoral activity through the inhibition of topoisomerase II and thus, prevents chain unfolding and separation in DNA replication as well as DNA repair. This ultimately leads to cell death by apoptosis [3]. Other mechanisms that might be involved are intercalation into DNA resulting in the inhibition of DNA synthesis and function, and the formation of cytotoxic reactive oxygen species (ROS) that results in single- and double-stranded DNA breaks with subsequent inhibition of DNA synthesis and function. On the other side, the benefits of doxorubicin may be hampered by its significant dose-related adverse effects, including cardiotoxicity, mainly in the form of cardiomyopathy, and congestive heart failure, myelosuppression, gastrointestinal distress, alopecia, stomatitis, etc., which lead to dose limitations in doxorubicin use [4,5,6].

Hyperthermia has been recently introduced as an adjuvant therapy for cancer and presents promising opportunities to treat cancers, especially in combination with chemotherapy or radiotherapy. Indeed, many clinical experiments have demonstrated that the addition of hyperthermia to radiotherapy or chemotherapy significantly improves tumor control and patient survival rates [7,8,9,10,11]. Hyperthermia is defined as a treatment in which the target tissue is exposed to high temperatures that either destroy the tissues directly (thermal ablation with temperatures above 47 °C) or render the cancer cells more susceptible to other treatment modalities (thermal sensitization in the temperature ranges of 41–45 °C). The commonly used heating methods are based on limb perfusion, incubation chambers, lasers, microwaves, radiofrequency or ultrasound thermal ablation, implantable ferromagnetic needles, etc. [12] Magnetic hyperthermia is a promising method for site-specific heating that reaches deeper tissue, in which magnetic nanoparticles (MNPs) play an important role by relaying the externally delivered high frequency alternating magnetic field (AMF) [13]. Indeed, direct injection of MNPs into solid tumors, followed by AMF exposure, has been shown to result in tumor regression [14,15,16,17,18]. However, conventional hyperthermia methods, including standard magnetic hyperthermia, do not discriminate between the target and the surrounding normal tissues, and this non-selective tissue heating can lead to serious side effects. These drawbacks must be overcome by the development of an efficient, targeted hyperthermia method. For this purpose, magnetic remote actuation of intra-lysosomal heat generation has been recently proposed as a means of eradicating cancer cells. Indeed, we and others have shown that intracellular or intra-lysosomal hyperthermia induced by targeted MNPs, which have been internalized and accumulated in the lysosomes of cancer cells, can specifically kill cancer cells without damaging healthy surrounding cells [19,20,21,22,23]. Indeed, in these studies, no perceptible temperature rise in the cell culture medium occurred during AMF exposure. We elaborated a nanoplatform, termed Gastrin–MNP, which is composed of an iron oxide MNP coated with polyethylene glycol-amine and decorated with (i) a synthetic replicate of gastrin, which binds specifically to the gastrin receptor (CCK2R), a receptor overexpressed in endocrine tumors, and allows MNPs internalization in cells overexpressing this receptor; (ii) a fluorescent label DY647 [22]. Gastrin–MNPs recognized the endocrine tumoral cells INR1G9 expressing the CCK2R (INR1G9-CCK2R) with high specificity that is subsequently internalized using the CCK2R-dependent signaling and finally accumulated in the lysosomes. We showed that the internalization of a minute amount of Gastrin–MNPs (1–2 pg Fe/cell) induced the death of ~30% of INR1G9-CCK2R cells upon AMF exposure (40 mT, 275 kHz) without affecting the viability of cells devoid of MNPs [22,23,24]. Moreover, we showed that cell death is initiated in the lysosomes, consecutively to a significant temperature increase in the immediate vicinity of the MNPs in response to AMF application without increasing the temperature at the outer surface of lysosomes and is mediated through a lysosomal death pathway [22,23]. Thus, this approach that we called magnetic intra-lysosomal hyperthermia (MILH), differs from standard magnetic hyperthermia whereby tumor eradication is achieved with large doses of MNPs, which cause the temperature elevation of the whole tumor.

The aim of this study was to analyze whether the combination of MILH with chemotherapy can increase the efficiency of cancer cell eradication. Endocrine tumoral cells were incubated with Gastrin–MNPs, treated with doxorubicin, the drug commonly used to treat endocrine tumors, and exposed to AMF. The impact of combined treatments was analyzed on cell viability and cell death. The molecular mechanisms of cell death were also studied. Here, we report that the combination of MILH with doxorubicin increases the eradication efficiency of endocrine tumor cells with synergism. We also demonstrate that these two treatments activate two different cell death pathways that respectively depend on Caspase-1 and Caspase-3 activation.

## 2. Materials and Methods

### 2.1. Chemical Reagents

The synthesis method and characterization of the magnetic nanoplatform used in the present article have been previously described [22]. Briefly, the nanoplatform (Gastrin–MNP) is composed of commercial iron oxide MNPs coated with polyethylene glycol-amine (Gecco Dots, Lund, Sweden) and decorated with 100 molecules of a synthetic replicate of gastrin (Covalab, Villeurbanne, France) and 20 molecules of the fluorescent label NHS-DY647-PEG1 (Dyomics GmbH, Jena, Germany). The size of the magnetic core determined by transmission electron microscopy was 8.7 ± 1.6 nm. The specific absorption rate of these MNPs is 13 W/g at 275 kHz and 40 mT. Doxorubicin and MTT [(3-(4,5-dimethylthiazol-2-yl)-2,5-diphenyltetrazolium bromide] were purchased from Sigma-Aldrich (Saint-Quentin-Fallavier, France).

### 2.2. Cell Culture

The glucagon-producing hamster tumoral cell line INR1G9, stably expressing the CCK2R (INR1G9-CCK2R), was obtained as previously described [25] and cultured in RPMI 1640 medium containing 10% heat inactivated fetal bovine serum (FBS) and 100 units/mL penicillin/streptomycin (Life technologies, Courtaboeuf, France). Cells were grown in a humidified atmosphere at 95% air and 5% CO_2_ at 37 °C.

### 2.3. Gastrin–MNP Localization in Lysosomes by Confocal Microscopy

Cells were plated onto Cellview culture dishes (Greiner Bio-One, Courtaboeuf, France). After overnight growth, cells were incubated with Gastrin–MNPs ([Fe] = 16 μg/mL) in incubation medium (RPMI 1640 buffered with 10 mM HEPES buffer pH 7.4 containing 0.5% heat inactivated FBS and 100 units/mL penicillin-streptomycin) for 24 h. For lysosome staining, cells were incubated for 15 min in the presence of 75 nM LysoTracker Red DND-99 (excitation: 570 nm, Life technologies). Gastrin–MNP (excitation: 633 nm) and Lysotraker co-localization was analyzed using a LSM780 confocal microscope (Zeiss, Marly le Roi, France).

### 2.4. Cell Exposure to Alternating Magnetic Field

Cell treatment with a high frequency alternating magnetic field (AMF: 275 kHz, 40 mT) was performed as follows: cells were seeded 24 h before the experiments onto four-compartment Cellview dishes (Greiner Bio-One) at a density of 60 × 10^3^ cells/compartment, grown overnight and incubated with Gastrin–MNPs for 24 h at 37 °C in incubation medium (RPMI 1640 buffered with 10 mM HEPES buffer pH 7.4 containing 0.5% heat inactivated FBS and 100 units/mL penicillin-streptomycin) to allow MNP internalization and accumulation in lysosomes. The incubation medium was removed and cells were rinsed twice with incubation medium. Cells were then incubated or not with doxorubicin in incubation medium (500 µL per well) for 24 h. Finally, cells were exposed or not to AMF delivered by a magnetic inductor (Fives Celes, Lautenback, France) for 2 h as previously described [22]. The temperature of the incubation medium was strictly maintained at 37.0 ± 0.4 °C and controlled using a thermal probe (Reflex, Neoptix, Quebec City, QC, Canada) placed in the incubation medium. At the end of the experiments, cells were placed in a humidified atmosphere at 5% CO_2_ at 37 °C for further analyses.

### 2.5. Cell Viability Analysis

A total of 1 × 10^4^ INR1G9-CCK2R cells was seeded in a 96-well plate, grown overnight and treated with increased concentrations of doxorubicin or Gastrin–MNPs in incubation medium for 24 or 48 h. Ten microliters of MTT (3-(4,5-dimethylthiazolyl-2)-2,5-diphenyltetrazolium bromide) at 5 mg/mL were added to each well and incubated for 2 h at 37 °C. After incubation, the cell culture medium was removed, and 100 µL of dimethylsulfoxid were added to each well and incubated for 1 h at 37 °C to dissolve the formed formalin crystals. After incubation, the absorbance (OD) values were measured at 570 nm. The cells maintained in the incubation medium without Gastrin–MNPs or doxorubicin served as negative controls.

The effects of AMF exposure on cell viability were investigated as follows: cells were seeded onto four-compartment Cellview dishes, incubated or not with Gastrin–MNPs in incubation medium, washed, incubated or not with doxorubicin and exposed to AMF as previously described. Twenty-four hours after AMF exposure, 20 µL of MTT at 5 mg/mL were added to each well of INR1G9-CCK2R cell culture and incubated for 2 h at 37 °C. After incubation, the cell culture medium was removed and 400 µL of dimethylsulfoxid were added to each well and incubated for 1 h at 37 °C to dissolve the formed formalin crystals. After incubation, the absorbance (OD) values were measured at 570 nm. The cells maintained in the incubation medium without MNPs or doxorubicin and not exposed to AMF served as negative controls.

### 2.6. Cell Death

The effects of AMF exposure on cell death were investigated as follows: cells were seeded onto four-compartment Cellview dishes, incubated with Gastrin–MNPs in incubation medium, washed, incubated with doxorubicin and exposed to AMF as previously described. Four hours after AMF exposure, dead cells were labeled with FITC-annexin V (AnnV) and/or propidium iodide (PI) (Cell Meter Annexin V apoptosis assay kit, AAT Bioquest, Sunnyvale, CA, USA) in accordance with the manufacturer’s instructions. The counting of labeled cells was carried out through the analysis of confocal microscopy images (LSM510, Zeiss) representing populations of 2000–3000 cells/experiment using Image J software (National Institutes of Health, Bethesda, MD, USA, https://imagej.nih.gov/ij/, 1997–2016).

### 2.7. Synergism/Additivity Analysis by the Chou–Talalay Method

Two doses of Gastrin–MNPs and three doses of doxorubicin were used, covering their IC_50_ for single treatment analysis. For combination studies, Gastrin–MNPs were used with 16 µg/mL of Fe; doxorubicin was used at 1, 3 and 5 µM. Percentages of inhibition of cell viability and induction of cell death were measured in the presence or absence of AMF exposure. The combination index (CI) was determined from the CI-isobologram method using CompuSyn software (ComboSyn, Inc., Paramus, NJ, USA) [26]. CI < 1 and CI = 1 indicated synergism and additivity, respectively [27].

### 2.8. Analysis of Caspase-1 and Caspase-3 Activation

Four hours after AMF exposure, cells were incubated with Fluorochrome-Labeled Inhibitors of Caspase-1 (FLICA, FAM-YVAD-FMK, excitation: 488 nm) or Caspase-3 (FLICA, FAM-DEVD-FMK, excitation: 488 nm) reagent (Immunochemistry technologies, Bloomington, MN, USA) in accordance with the manufacturer’s instruction. Next, cells were washed and analyzed by confocal microscopy (LSM510, Zeiss). The counting of labeled cells was carried out by analyzing confocal microscopy images representing populations of 2000–3000 cells/experiment using Image J software.

### 2.9. Statistical Analysis

Results are expressed as the mean ± SEM of at least three independent experiments. The statistical analysis was performed using ANOVA tests. Differences were considered significant when *p* < 0.05.

## 3. Results

### 3.1. Doxorubicin and Gastrin–MNP Cytotoxicity

**Figure 1:**
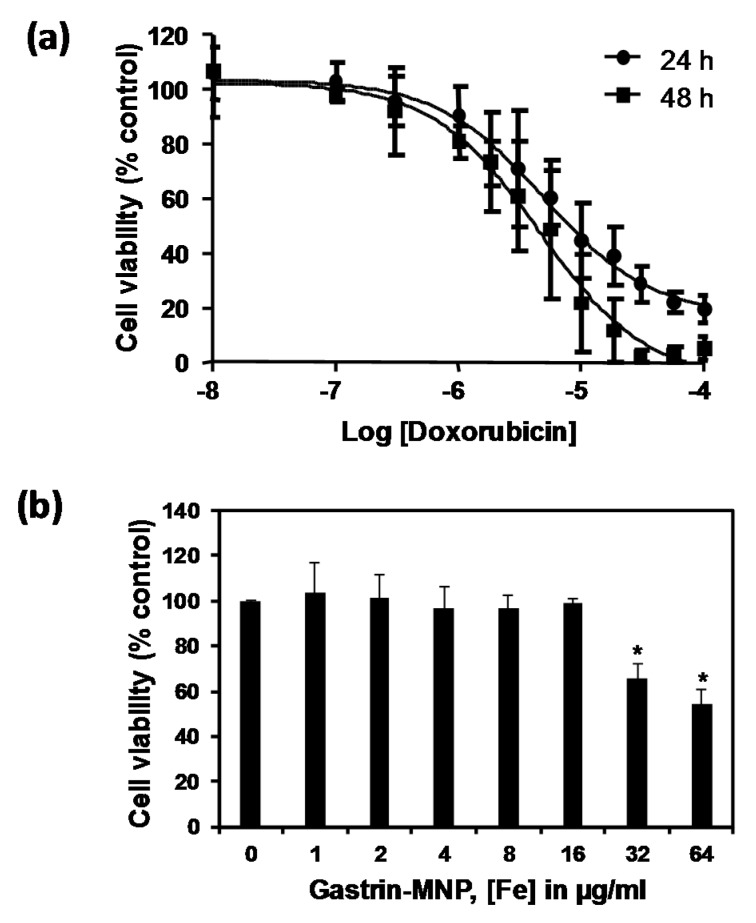

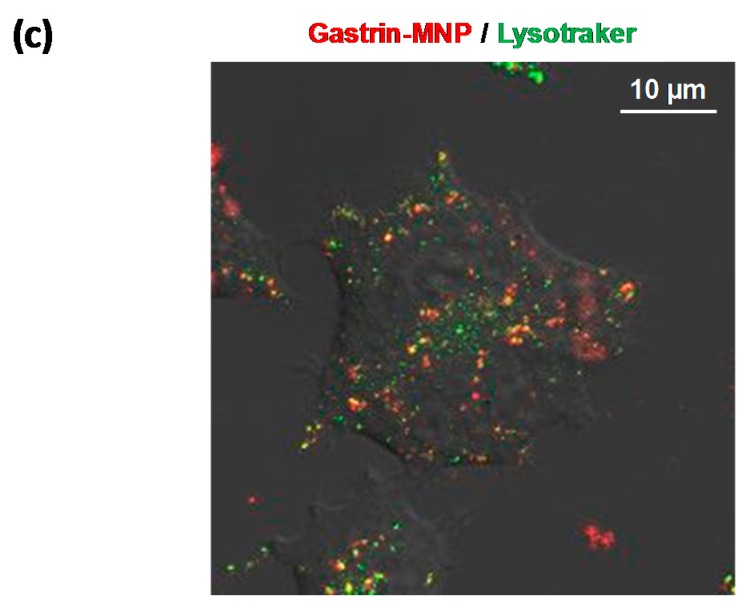
(**a**) Cytotoxicity of doxorubicin. INR1G9-CCK2R cells were incubated with increased concentrations of doxorubicin for 24 and 48 h. Cell viability was analyzed by MTT assay. Error bars represent the SEM of four independent experiments; (**b**) The cytotoxicity of Gastrin–MNPs (magnetic nanoparticles). INR1G9-CCK2R cells were incubated with increased concentrations of Gastrin–MNPs for 48 h. Cell viability was analyzed by MTT assay. The error bars represent the SEM of four independent experiments. The stars represent significant decrease of cell viability over control cells in absence of Gastrin-MNP; (**c**) Localization of Gastrin–MNPs. INR1G9-CCK2R cells were incubated with Gastrin–MNPs ([Fe] = 16 µg/mL) for 24 h. Lysotraker (75 nM) was added 15 min before analysis by confocal microscopy. Representative image illustrating lysosome occupancy by Gastrin–MNPs.

The doxorubicin cytotoxicity was measured on endocrine tumoral INR1G9-CCK2R cells at two incubation times (24 and 48 h). The analysis of the doxorubicin dose-response using MTT assays showed that doxorubicin has an inhibitory dose-dependent effect on the viability of INR1G9-CCK2R cells. The IC_50_ values of doxorubicin were 5.0 ± 0.1 and 4.6 ± 0.1 µM at 24 and 48 h of incubation, respectively (Figure 1a).

We also assessed the cytotoxicity of the Gastrin–MNPs. Figure 1b shows that, when INR1G9-CCK2R cells were incubated with Gastrin–MNPs at Fe concentrations ranging from 1 to 16 µg/mL and left for 48 h in contact with cells, the viability of INR1G9-CCK2R cells was above 98.8 ± 2.0%, indicating that Gastrin–MNPs were not toxic. At higher concentrations, Gastrin–MNPs were cytotoxic to INR1G9-CCK2R cells. Indeed, Gastrin–MNPs at Fe concentrations of 32 and 64 µg/mL decreased the cell viability by 34.0 ± 5.9% and 45.6 ± 6.3%, respectively.

Finally, we showed that Gastrin–MNPs are mainly localized in the lysosomal compartment of INR1G9-CCK2R cells after 24 h of incubation (Figure 1c).

**Figure 2:**
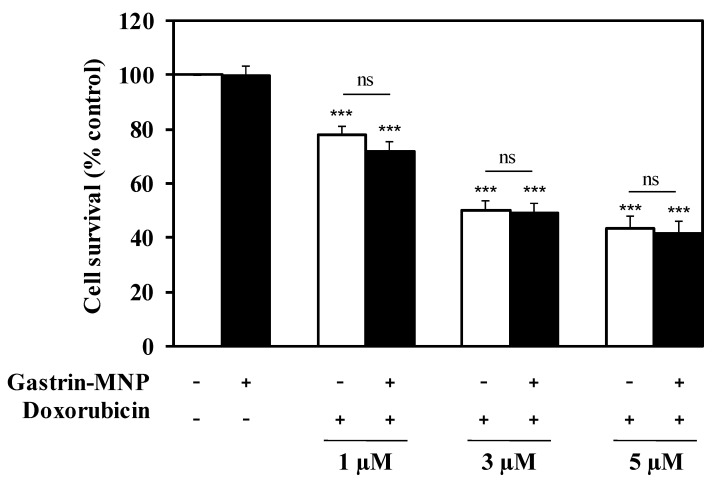
Gastrin–MNPs did not modify doxorubicin cytotoxicity on INR1G9-CCK2R cells. Cells were pre-incubated with Gastrin–MNPs ([Fe] = 16 µg/mL) for 24 h, washed to eliminate unbound nanoparticles and then incubated with different concentrations of doxorubicin for another 24 h. Cell viability was analyzed by MTT assay. Each value represents the mean ± SEM of at least four independent experiments. The stars represent significant decrease of cell viability over control cells in absence of doxorubicin and Gastrin-MNPs.

Subsequently, we examined the cytotoxicity of doxorubicin in the presence of Gastrin–MNPs. INR1G9-CCK2R cells were incubated with Gastrin–MNPs ([Fe] = 16 µg/mL) for 24 h before the addition of different concentrations of doxorubicin for another 24 h (Figure 2). In agreement with the previous results, the incubation of Gastrin–MNPs at a Fe concentration of 16 µg/mL did not cause cytotoxic effects on INR1G9-CCK2R cells. Moreover, the cytotoxicity of doxorubicin was not significantly different in the presence or absence of Gastrin–MNPs. For example, 5 µM of doxorubicin inhibited the INR1G9-CCK2R cell viability by 58.4 ± 4.2% in the presence of Gastrin–MNPs, compared to 56.7 ± 4.4% in the absence of Gastrin–MNPs. Similar results were obtained with lower concentrations of doxorubicin. These results indicate that the non-cytotoxic concentration of Gastrin–MNPs did not increase the cytotoxicity of doxorubicin. The Gastrin–MNPs concentration at 16 µg/mL of Fe, which corresponds to the maximal dose that is devoid of a cytotoxic effect, was then used throughout the study.

### 3.2. Effects of the Combination of Magnetic Intra-Lysosomal Hyperthermia and Doxorubicin Treatments

#### 3.2.1. Cell Viability Analysis

**Figure 3:**
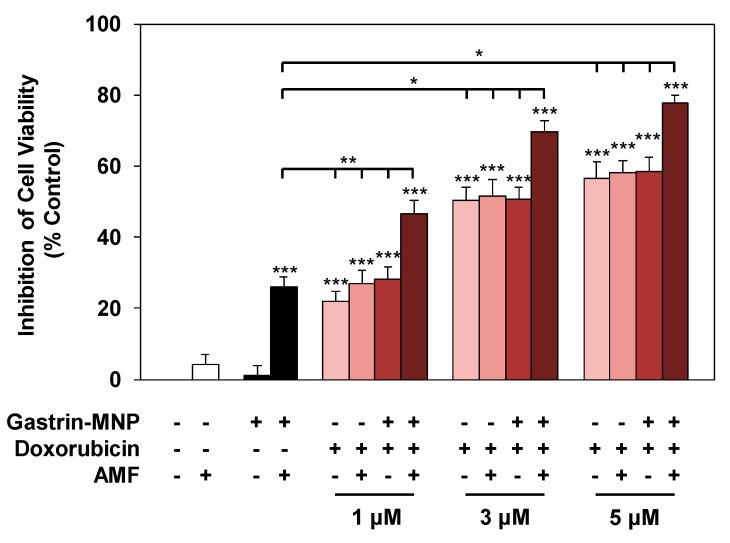
Effect of the combination of magnetic intra-lysosomal hyperthermia and doxorubicin treatments on cell viability. INR1G9-CCK2R cells were incubated with Gastrin–MNPs ([Fe] = 16 µg/mL) for 24 h, allowing their internalization and lysosome accumulation and washed to eliminate unbound and non-internalized nanoparticles, before the addition of different concentrations of doxorubicin for another 24 h and exposure or not to AMF (40 mT, 275 kHz). The impact of the treatments on cell viability was determined 24 h after AMF application by MTT assay. Significant differences (*) compared to the control condition corresponding to cells devoid of Gastrin–MNPs in the absence of AMF exposure and doxorubicin treatment is indicated above the histogram bar. Statistical significance (*) between other conditions is also indicated. Results are the mean ± SEM of five separate experiments.

The effect of the association of doxorubicin and Magnetic Intra-Lysosomal Hyperthermia (MILH), induced through the application of AMF to cancer cells that have accumulated MNPs inside their lysosomes, was studied on cell viability. Doxorubicin treatments for 24 or 48 h inhibited the viability of INR1G9-CCK2R cells with approximately the same efficacy (Figure 1a). We previously showed that Magnetic Intra-Lysosomal Hyperthermia (MILH) decreased the viability of INR1G9-CCK2R cells 24 and 48 h after AMF exposure [22,23]. To analyze the effect of treatment combination, INR1G9-CCK2R cells were incubated with Gastrin–MNPs for 24 h to allow their internalization and accumulation in the lysosomes (Figure 1c) and washed to eliminate unbound and non-internalized MNPs, before the addition of different concentrations of doxorubicin for 24 h. Cells were then exposed to AMF (40 mT, 275 kHz), and the impact of the treatments on cell viability was analyzed 24 h after AMF exposure. Figure 3 shows that the combination of MILH and doxorubicin treatments decreased the cell viability more efficiently than each individual treatment. Indeed, AMF exposure inhibited the viability of INR1G9-CCK2R cells containing Gastrin–MNPs (MILH) and incubated with 5 µM of doxorubicin by 77.7 ± 2.1%, whereas single treatment by MILH or 5 µM of doxorubicin decreased the cell viability by 26.1 ± 2.8% and 56.7 ± 4.4%, respectively. Identical results were obtained with lower doses of doxorubicin. Of note, AMF exposure neither affected the viability of control cells devoid of MNPs, nor modified the cytotoxicity of doxorubicin, whatever the doxorubicin concentration used. Doxorubicin treatment at the concentrations of 1, 3 or 5 µM decreased the viability of INR1G9-CCK2R cells by 26.9 ± 3.7%, 51.7 ± 4.6% and 58.0 ± 3.6% in the presence of AMF application, relative to 21.9 ± 3.0%, 50.2 ± 3.8% and 56.7 ± 4.4% in the absence of AMF exposure. As expected, Gastrin–MNPs at a concentration of 16 µg/mL of Fe neither affected the viability of INR1G9-CCK2R, nor increased doxorubicin cytotoxicity, in the absence of AMF. Indeed, while 1, 3 or 5 µM of doxorubicin decreased the viability of INR1G9-CCK2R cells by 28.3 ± 3.4%, 50.7 ± 3.4% and 58.4 ± 4.2% in the presence of Gastrin–MNPs, the same concentrations of doxorubicin inhibited it by 21.9 ± 3.0%, 50.2 ± 3.8% and 56.7 ± 4.4% in the absence of Gastrin-MNPs and AMF application. Therefore, these results indicate that the association of MILH with doxorubicin-induced cytotoxicity eradicates tumoral cells with a higher efficiency than the single treatments.

#### 3.2.2. Cell Death Study

**Figure 4:**
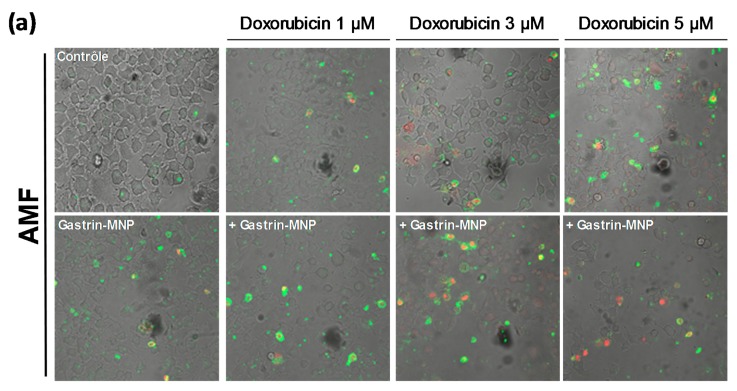

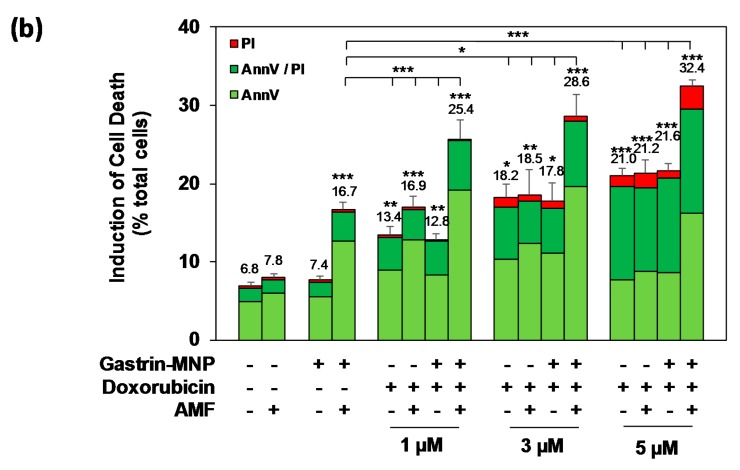
Effects of the combination of Magnetic Intra-Lysosomal Hyperthermia and doxorubicin treatments on cell death. INR1G9-CCK2R cells were incubated with Gastrin–MNPs ([Fe] = 16 µg/mL) for 24 h, allowing their internalization and lysosome accumulation and washed to eliminate unbound and non-internalized nanoparticles, before the addition of different concentrations of doxorubicin for another 24 h and exposed or not to AMF (40 mT, 275 kHz). The impact of the treatments was determined 4 h after AMF exposure by counting cells labeled with FITC-tagged annexin V (AnnV) and/or propidium iodide (PI) which identified dead cells. PI staining detected necrotic cells and AnnV and AnnV/PI labeling revealed regulated cell death including apoptosis and related mechanisms of cell death. (**a**) Representative images illustrating AnnV/PI labeling. (**b**) Quantification of AnnV/PI labeling. The percentages of induction of cell death are indicated above the histogram bars. Significant differences (*) compared to the control condition corresponding to cells devoid of Gastrin–MNPs in absence of AMF exposure and doxorubicin treatment are indicated above histogram bars. Statistical significance (*) between other conditions is also indicated. A quantity of 2000–3000 cells/experiment was analyzed and the results are the mean ± SEM of five separate experiments.

The impact of the association of MILH with doxorubicin was also analyzed in relation to the induction of cell death. According to the literature, the impact of doxorubicin on cell death is usually measured by annexin V and/or propidium iodide labeling after 4 to 24 h of incubation. We previously showed that cell death induced by MILH can be detected 4 h after AMF exposure [22,23]. To analyze the effect of treatments combination on cell death, INR1G9-CCK2R cells were incubated with Gastrin–MNPs for 24 h and washed to eliminate unbound and non-internalized MNPs, before the addition of different concentrations of doxorubicin for 24 h. Cells were then submitted or not to AMF, and the impact of the treatments was determined by counting dead cells labeled with FITC-tagged annexin V and/or propidium iodide (AnnV/PI) 4 h after AMF exposure. The Figure 4 showed that the combination of MILH and doxorubicin treatments caused cell death. Moreover, the MILH/doxorubicin combination increased the cell death rate more efficiently than individual treatments. Indeed, AMF exposure induced the death of 32.4 ± 0.8% of INR1G9-CCK2R cells containing Gastrin–MNPs (MILH) and incubated with 5 µM of doxorubicin, whereas individual treatments were less efficient as they induced the death of 16.7 ± 1.0% and 21.0 ± 1.0%, respectively. Identical results were obtained with lower doses of doxorubicin (Figure 4). Interestingly, AnnV or AnnV/PI staining, which mainly labels dead cells in these treatments, indicated that cell death does not match with necrosis but involves apoptosis or a related mechanism of cell death, such as pyroptosis [28,29]. As expected, AMF exposure neither affected the viability of control cells devoid of MNPs, nor modified the cytotoxicity of doxorubicin, whatever the doxorubicin concentration used. Likewise, Gastrin–MNPs neither induced the death of INR1G9-CCK2R cells, nor increased the death rate induced by doxorubicin, in the absence of AMF exposure. All together, these results indicate that the induction of cell death by the combination of MILH and doxorubicin was higher than that of individual treatments, in agreement with previous results obtained for cell viability (Figure 3).

#### 3.2.3. Synergism/Additivity Analysis

We also examined a possible synergistic/additive interaction between MILH and doxorubicin on cell viability and cell death using the Chou–Talalay method [26,27]. Three doses of doxorubicin (1, 3 and 5 µM) and a non-cytotoxic Gastrin–MNPs concentration ([Fe] = 16 µg/mL) were used to determine whether a synergistic/additive anti-tumoral interaction was present between the two compounds. The combination index (CI) was determined from the CI-isobologram method [26,27]. Values representing the inhibition of cell viability, the induction of cell death and their combination indexes (CI) are presented in Table 1. CI values indicated a synergistic or an additive effect on the inhibition of cell viability when 1 or 3 µM of doxorubicin is combined with MILH. The CI values analysis on cell death induction showed a synergistic effect of the combination MILH with the three doses of doxorubicin. Therefore, these results indicate that the association of MILH with doxorubicin-induced cytotoxicity not only increases the eradication efficiency of tumoral cells relative to single treatments but also that this combination acts with synergism or additivity to decrease tumoral cell viability or induce cell death. Then, we analyzed the mechanisms of cell death under the different conditions.

### 3.3. Mechanism of Cell Death: Analysis of Capsase-1 and Caspase-3 Activation

**Figure 5:**
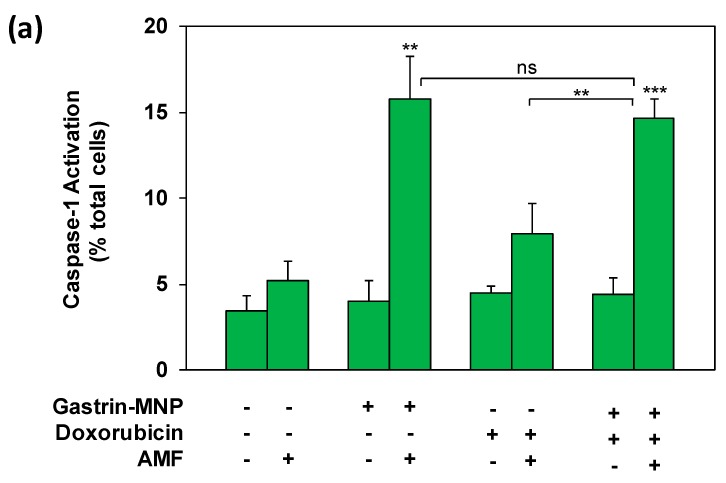

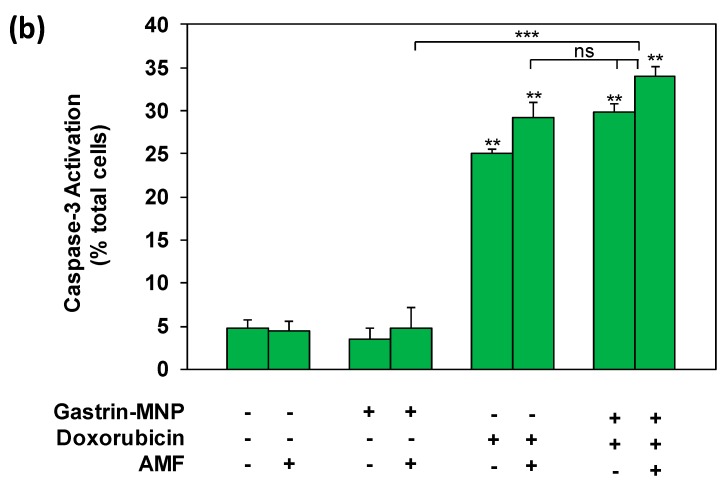
Analysis of Caspase-1 and Caspase-3 activation. INR1G9-CCK2R cells were incubated with Gastrin–MNPs ([Fe] = 16 µg/mL) for 24 h, allowing their internalization and lysosome accumulation and washed to eliminate unbound and non-internalized nanoparticles, before the addition of 3 µM of doxorubicin for another 24 h and exposure or not to AMF (40 mT, 275 kHz). The activation of Caspase-1 (**a**) and Caspase-3 (**b**) was analyzed 4 h after AMF exposure by confocal microscopy of cells labeled with FAM-FLICA-Casp1 or FAM-FLICA-Casp3. Results are expressed in % of total cells labeled with fluorescent Caspase substrate and are the mean ± SEM of at least three separate experiments. Significant differences (*) compared to the control condition corresponding to cells devoid of Gastrin–MNPs in absence of AMF exposure and doxorubicin treatment are indicated above histogram bars. Statistical significance (*) between other conditions is also indicated.

We previously demonstrated that MILH induces the death of cancer cells through a non-apoptotic pathways that is triggered inside lysosomes through the catalysis of Fenton reaction-enhancing ROS production, promoting Cathepsin-B release from lysosomes into the cytosol and activating Caspase-1-dependent cell death [23]. Doxorubicin was shown to induce apoptotic cell death which depends on Caspase-3 activation [3]. We analyzed the mechanism of cell death induced in the MILH/doxorubicin combined treatments, by measuring the activation of Caspase-1 and Caspase-3, 3 h after AMF exposure. Figure 5a shows that the combination of MILH and doxorubicin treatment significantly increased the Caspase-1 activation by 4.2 ± 0.3-fold (positive cells: 14.6 ± 1.1% versus 3.5 ± 0.9% in control cells), whereas MILH alone increased it by 4.5 ± 0.7-fold (positive cells: 15.8 ± 2.4% versus 3.5 ± 0.9% in control cells) and doxorubicin did not significantly activate Caspase-1 (positive cells: 7.9 ± 1.8%). Moreover, the level of Caspase-1 activation was not significantly different between the MILH/doxorubicin combination and MILH individual treatment, indicating that cell death induced through Caspase-1 activation in the combined treatment was related to the MILH effect.

As shown in Figure 5B, doxorubicin significantly increased Caspase-3 activation by 5.2 ± 1.4-fold (positive cells: 25.1 ± 9.4% versus 4.8 ± 0.3% in control cells), whereas MILH did not activate Caspase-3 (positive cells: 4.7 ± 0.6%) 3 h after AMF exposure. The combination of MILH and doxorubicin treatments significantly increased Caspase-3 activation by 7.1 ± 0.9-fold (positive cells: 34.0 ± 6.9% versus 4.8 ± 0.3% in control cells). However, this effect was not significantly different to that of doxorubicin individual treatment (positive cells: 25.1 ± 9.4%), indicating that doxorubicin was responsible for Caspase-3 activation in the combined treatment.

All together, these results indicate that MILH and doxorubicin separately induce the death of cancer cells by two different mechanisms, corresponding to the activation of a non-apoptotic and an apoptotic pathways depending on Caspase-1 and Caspase-3 activation, respectively. Moreover, the synergetic/additive effect of the MILH/doxorubicin combination results from the activation of both of these cell death pathways which depends on Caspase-1-dependent cell death and apoptosis.

## 4. Discussion

Nowadays, conventional cancer therapies include surgery, radiotherapy and/or chemotherapy. Several studies have provided evidence that hyperthermia could enhance the efficiency of radiotherapy/chemotherapy or overcome drug resistance in many cancer cells [30,31,32]. This rationale is based on the direct killing effect of cancer cells at temperatures above 41/42 °C [33] and various chemotherapeutic drugs present a thermal sensitization effect corresponding to an increased sensitivity of cancer cells to chemotherapeutic agents at these temperatures. For example, hyperthermia using a water bath was shown to sensitize breast cancer cells to bortezomid or TNF (Tumor Necrosis Factor), resulting in enhanced cell death [30,34]. Although in vitro studies of hyperthermia with chemotherapeutic drugs have shown promising results, its translation to the clinic presents limitations in its application as a treatment modality. These include the avoidance of non-specific damage to adjacent tissues, the occurrence of tachycardia and malaise and detrimental impacts on tissue metabolism, blood flow, organ function and tissue repair [32,35]. For these reasons, alternative hyperthermia application approaches are being actively pursued. The use of MNPs for localized thermal anti-tumoral therapy is a novel and attractive strategy [36]. The first approach, which has been developed, is commonly named magnetic fluid hyperthermia. It takes advantage of the deposition of thermal energy by MNPs under an applied AMF, resulting in local heating of the tumor [37]. Magnetic fluid hyperthermia has been shown to increase the efficacy of several chemotherapeutic drugs. As an example, magnetic hyperthermia using iron oxide MNPs enhanced the efficacy of cisplatin, bortezomib or doxorubicin, in the destruction of cancer cells [38,39,40]. Interestingly, chemotherapeutic drugs, such as cisplatin or bortezomib, are more effective at reducing cell viability when combined with magnetic fluid hyperthermia than with hyperthermia using a hot water bath [39,40]. However, magnetic fluid hyperthermia presents several limitations for translation to clinic, although in vitro studies have shown promising results. The first limitation is the concentration (~10 mg/mL) of MNPs necessary to induce tumoral tissue hyperthermia when submitted to AMF, indicating that only intra-tumoral injection of a high concentration of MNPs is effective for increasing the temperature of tumoral tissues. The other limitations are the adverse effects due to temperature increase, such as damage to adjacent tissues, the occurrence of tachycardia and malaise and impacts on tissue metabolism, blood flow, organ function, etc. Here, we have demonstrated, for the first time, that Magnetic Intra-Lysosomal hyperthermia (MILH) that occurs without inducing any perceptible temperature rise increases the efficacy cancer cells eradication when combined with the chemotherapeutic drug, doxorubicin. Using gastrin-grafted nanoparticles (Gastrin–MNPs) which recognize cells expressing the CCK2R and subsequently internalize using CCK2R-dependent signaling [22], we previously demonstrated that Gastrin–MNPs accumulate in the lysosomes and kill cancer cells through MILH, which occurs with a significant temperature increase at the immediate vicinity of the MNPs without increasing the temperature at the outer surface of lysosomes or the incubation medium [23]. Here, we studied whether the combination of MILH with a chemotherapeutic drug could increase the eradication efficiency of cancer cells. After determining the IC_50_ of doxorubicin, which is one of the commonly used anti-cancer drugs to treat pancreatic endocrine tumors, we combined this dose and lower doses of doxorubicin with MILH and analyzed the effect on cell death and cell survival. We showed that the association of MILH with doxorubicin-induced cytotoxicity decreased the viability and increased the death rate of cancer cells with a higher efficiency than single treatments. Moreover, we demonstrated that this combination acts with synergism or additivity to eradicate cancer cells. Finally, we showed that the synergistic/additive effect of the MILH/doxorubicin combination results from the activation of two different cell death pathways induced by each single treatment, which depends on Caspase-1-dependent cell death and Caspase-3-dependent apoptosis, respectively. Therefore, these results are not only in agreement with numerous studies showing that hyperthermia enhances the efficiency of chemotherapy [30,31,32], but they also demonstrate that a temperature increase restrained within the lysosomal compartment also increases the sensitization of cancer cells to chemotherapy. This synergistic interaction between heat and cytostatic/cytotoxic treatments has been already observed in preclinical studies but not in conditions presenting a temperature increase restrained to the lysosome compartment [41,42]. Our findings will result in the development of new types of anti-tumoral, intra-lysosomal-thermo/chemotherapy that might have much better curative effects compared to chemotherapy alone and devoid of adverse effects linked to standard hyperthermia approaches.

## 5. Conclusions

In conclusion, our results indicate that the intracellular accumulation of magnetic nanoparticles followed by doxorubicin treatment and exposure to a high frequency alternating magnetic field might provide a promising approach in the treatment of patients without inducing adverse effects of others hyperthermia approaches, such as hot water, magnetic fluid hyperthermia, etc. Many studies have provided evidence that hyperthermia enhances the efficiency of chemotherapy or overcomes drug resistance in many cancer cells. Here, we demonstrated, for the first time, that the association of Magnetic Intra-Lysosomal Hyperthermia (MILH) application, which occurs without inducing any perceptible temperature rise, with doxorubicin-induced cytotoxicity increases the efficiency of the eradication of cancer cells. Moreover, we showed that the combination of both treatments acts with synergism to kill cancer cells. This will result in the development of new types of anti-tumoral, intra-lysosomal thermo/chemotherapy that might have much better curative effects compared to chemotherapy alone and are devoid of adverse effects linked to standard hyperthermia approaches. These findings also suggest that MILH can decrease the required dose of chemotherapeutic drugs, such as doxorubicin, and thereby reduce their side effects. Consequently, it might be possible to reduce the clinically effective dosage of doxorubicin by administering it in combination with MILH, thereby potentially reducing the incidence of doxorubicin-related side effects.

## Figures and Tables

**Table 1:**
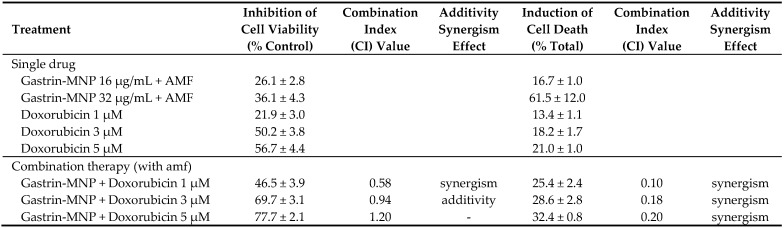
Analysis of combination treatments of Magnetic Intra-Lysosomal Hyperthermia (MILH) and doxorubicin. Gastrin–MNPs were used at a non-cytotoxic concentration ([Fe] = 16 µg/mL) and doxorubicin was used at 1, 3 and 5 µM. The percentage of inhibition of cell viability and induction of cell death in the presence or absence of AMF exposure are indicated. The combination index (CI) was determined from the CI-isobologram method using CompuSyn software [26]. CI < 1 and CI = 1 indicate synergism and additivity, respectively [27].
